# The Impact of the Withdrawal of SGLT2 Inhibitors on Clinical Outcomes in Patients with Heart Failure

**DOI:** 10.3390/jcm13113196

**Published:** 2024-05-29

**Authors:** Masaki Nakagaito, Teruhiko Imamura, Ryuichi Ushijima, Makiko Nakamura, Koichiro Kinugawa

**Affiliations:** Second Department of Internal Medicine, University of Toyama, Toyama 930-0194, Japan; mgaito128@gmail.com (M.N.); ryuryu0702@gmail.com (R.U.); nakamuramk1979@gmail.com (M.N.); kinugawa0422@gmail.com (K.K.)

**Keywords:** heart failure, SGLT2 inhibitor, hospitalization, medical cost

## Abstract

**Background**: The clinical impact of the withdrawal of sodium–glucose cotransporter 2 inhibitors (SGLT2i) on all-cause readmission in patients with heart failure remains unknown. **Methods**: We enrolled a total of 212 consecutive patients who were hospitalized for heart failure and received SGLT2i during their index hospitalization between February 2016 and July 2022. Of these patients, 51 terminated SGLT2i during or after their index hospitalization. We evaluated the prognostic impact of the withdrawal of SGLT2i on the primary outcome, which was defined as the all-cause readmission rate/times. **Results**: Over a median of 23.2 months, all-cause readmission occurred in 38 out of 51 patients (74.5%) withdrawn from SGLT2i and 93 out of 161 patients (57.8%) with continuation of SGLT2i (*p* = 0.099). The incidence of all-cause readmissions per year was 0.97 [0–1.50] in patients withdrawn from SGLT2i and 0.50 [0–1.03] in patients with continuation of SGLT2i (*p* = 0.030). There was no significant difference in total medical costs (62,906 [502–187,246] versus 29,236 [7920–180,305] JPY per month, *p* = 0.866) between both patient groups. **Conclusions**: Termination of SGLT2i may be associated with incremental all-cause readmission and no benefit in reducing total medical costs.

## 1. Introduction

Heart failure (HF) is one of the most common cardiovascular diseases and is associated with frequent hospitalization and mortality [1]. Due to the aging of the overall population, the number of patients with HF is expected to increase in the near future. Treatment with sodium–glucose cotransporter 2 inhibitors (SGLT2i) improves cardiovascular outcomes in patients with HF, regardless of the presence or absence of diabetes mellitus (DM). Large-scale randomized controlled trials (RCTs) demonstrated that dapagliflozin and empagliflozin, two of the SGLT2is, improved the outcomes of patients with HF regardless of the patient’s left ventricular ejection fraction (LVEF) levels [2,3,4,5]. They are currently the only drugs that have been proven to improve the outcomes of patients with HF with preserved ejection fraction (HFpEF) in RCTs.

Furthermore, a recent RCT demonstrated that the risk of cardiovascular death or hospitalization for HF increased in patients withdrawn from empagliflozin [6]. This finding suggested a chronic effect of SGLT2i in patients with HF even after years of treatment, which dissipated after withdrawal of the drug. The immediate increase in the risk of cardiovascular events after the withdrawal of SGLT2i may be related to the inactivation of the acute effects of SGLT2i, such as anti-inflammatory, oxidative stress-reducing, and diuretic effects [7]. The finding that SGLT2i was effective in reducing HF events even in patients with recent worsening of heart failure supports this hypothesis [8]. However, the clinical impact of withdrawal of SGLT2i on non-cardiovascular disease in patients with HF remains unknown. Given its pleiotropic effects, including its kidney-protective effects, the withdrawal of SGLT2i may also increase the risk of non-cardiovascular disease [9,10]. Conversely, the withdrawal of SGLT2i may contribute to reducing the adverse effects associated with SGLT2i treatment, which may also have an impact on clinical outcomes. Furthermore, although several studies from different countries have evaluated the cost-effectiveness of SGLT2i for HF [11,12,13,14,15], the withdrawal of SGLT2i may show benefits in reducing total medical costs.

Thus, we compared the risk of hospitalization due to any cause between patients who discontinued SGLT2i and those who continued SGLT2i. In addition, we investigated the total medical costs associated with SGLT2i therapy for those patients.

## 2. Materials and Methods

This study was a single-center, prospective registry study designed to assess the efficacy of long-term SGLT2i therapy, which was initiated during the index hospitalization of patients with HF. In the present study, we retrospectively evaluated the hospitalization events and medical costs associated with SGLT2i in participants. This study secured approval from a local institutional ethics committee, which complied with the Declaration of Helsinki, and prior informed consent was diligently obtained from all individuals participating in this investigative endeavor.

### 2.1. Study Population

Consecutive patients who had been admitted for HF were involved in this study. HF was diagnosed according to the Framingham criteria. Most of the patients had New York Heart Association (NYHA) class III/IV symptoms upon admission. Participants received guideline-directed medical therapy for HF, including renin-angiotensin system inhibitors or angiotensin receptor–neprilysin inhibitors, beta-blockers, mineralocorticoid receptor antagonists, and diuretics, if applicable. We included patients receiving SGLT2i for the first time during their index hospitalization immediately following the stabilization of hemodynamics.

Patients were excluded from enrolment in this study if they met any of the following criteria: age <20 years, end-stage renal failure with estimated glomerular filtration rate (eGFR) <15 mL/min/1.73 m^2^, undergoing durable left ventricular assist device or heart transplantation, pregnancy or breastfeeding, current use of SGLT2i during the index hospitalization, and lost to follow-up within 90 days of the index hospitalization. In this study, patients who were hospitalized at an institute other than our own were also excluded for the accurate assessment of hospitalization events and costs. Adjustment of medical therapy was permitted as in real-world clinical practice.

### 2.2. Study Design

To investigate the long-term efficacy of SGLT2 in patients with HF, we compared the hospitalization events between the patients who discontinued SGLT2i after discharge and those who continued. Additionally, we compared the medical costs (expressed in Japanese yen (JPY) per month) between both patient groups to investigate the cost–benefits of SGLT2-incorporated medical therapy. Patients who discontinued SGLT2i during the index hospitalization for HF and those who discontinued SGLT2i during the observation period were assigned to the withdrawal group. Other patients continued SGLT2i after its initiation, and they were assigned to the continuation group. Because this study included patients admitted with decompensated HF, day 0 was defined as the time of index discharge to minimize selection bias. When patients died or were lost to follow-up, they were censored at that time. All patients were followed until the end of the observation period or for two years.

The primary clinical assessments were (1) the occurrence of all-cause hospitalization and (2) the total number of hospitalization events after index hospitalization. The hospitalization events included those for non-cardiovascular causes. The total number of hospitalization events was expressed in the number of hospitalizations per year. In addition, we assessed the total number of hospitalization events separately for cardiovascular and non-cardiovascular events. The secondary clinical assessments were (1) the recurrence of hospitalization for HF and (2) the total medical costs during the observation period. In this study, medical costs were defined as the summation of in-hospital medical costs and SGLT2i costs. For the withdrawal group, the cost of SGLT2i was added until the time when they were withdrawn.

### 2.3. Data Collection

Baseline characteristics including demographic, laboratory, and medication data at index discharge were retrieved. The estimated glomerular filtration rate (eGFR) was calculated using the guidelines from the Chronic Kidney Disease Epidemiology Collaboration. Standard echocardiographic findings during index hospitalization were retrieved. We defined HFrEF as left ventricular ejection fraction (LVEF) <40%. We defined DM as satisfying glycated hemoglobin (HbA1c) ≥6.5% or receiving antidiabetic treatment. The dose of loop diuretics was presented as furosemide equivalent. Among SGLT2i, the cost of canagliflozin 100 mg, dapagliflozin 10 mg, and empagliflozin 10 mg were 168 JPY per day, 264 JPY per day, and 188 JPY per day, respectively.

### 2.4. Statistical Analyses

All statistical computations were executed using JMP^®^ 15 (SAS Institute Inc., Cary, NC, USA). Significance was ascribed to outcomes featuring a two-sided *p*-value of <0.05. Continuous variables were conveyed as medians in conjunction with interquartile ranges, and the appropriate statistical assessments were conducted using the Wilcoxon test. Categorical data were articulated in terms of counts and corresponding percentages, and the appropriate statistical assessments were conducted using Pearson’s χ2 test.

The occurrence of all-cause hospitalization and the recurrence of hospitalization for HF were expounded upon using the Kaplan–Meier methodology, with inter-group disparities appraised via the log-rank test. Univariable and multivariable analyses with Cox proportional hazard models were performed to calculate the hazard ratio to assess the influence of various parameters on clinical outcomes. Univariable and multivariable linear regression analyses were performed to assess the influence of various parameters on the total number of hospitalization events. Variables significant with *p* < 0.050 in the univariable analyses were included in the multivariable analyses.

## 3. Results

### 3.1. Follow-Up and Patient Characteristics

**Figure 1:**
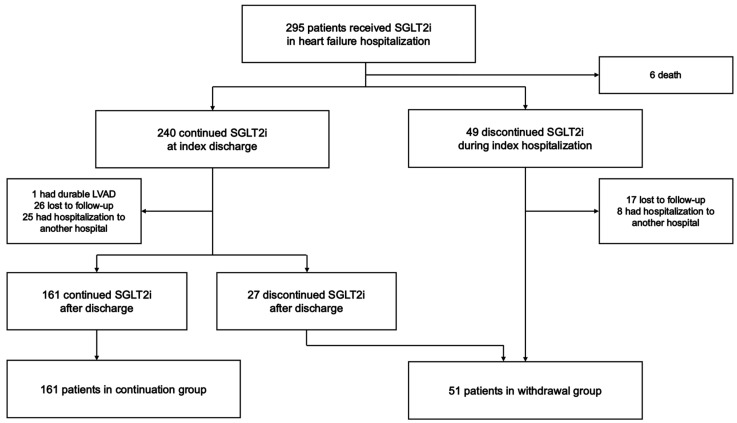
Patient flow chart.

From February 2016 to July 2022, 295 consecutive patients initiated SGLT2i during their hospitalization for HF. Of them, 6 died during hospitalization, 1 underwent implantation of a left ventricular assist device, 43 were lost to follow-up, and 33 were hospitalized at an institute other than our own after their index discharge. Finally, a total of 212 patients were included in the study (Figure 1).

Table 1 lists the patients’ baseline characteristics. The median age was 72 (66–81) years and 33% were women. HFrEF (LVEF < 40%) was noted in 84 patients (39%). DM was noted in 147 patients (69%). All patients with DM were those with type 2 DM. Baseline eGFR was 51.1 (38.2–64.2) mL/minute/1.73 m^2^. The baseline plasma BNP level was 142 (69–302) pg/mL. All diuretics were taken orally.

Of these, 161 patients continued SGLT2i during the observation period and were assigned to the continuation group. The other 51 patients terminated SGLT2i during or after their index hospitalization and were assigned to the withdrawal group. In the withdrawal group, the median duration that SGLT2i were continued was 17 (0–133) days. The reasons for SGLT2i withdrawal are listed in Supplementary Table S1. The most common reason for withdrawal of SGLT2i was urinary tract infection (*n* = 8, 15.7%). In contrast, the most common reason for withdrawal of SGLT2i during index hospitalization was fasting (*n* = 4).

Baseline characteristics between the two groups are compared in Table 1. Patients in the withdrawal group were older and underweight. The withdrawal group included more patients with higher BNP levels than the continuation group. There were more patients with DM in the withdrawal group than in the continuation group.

### 3.2. Primary Clinical Assessments

**Figure 2:**
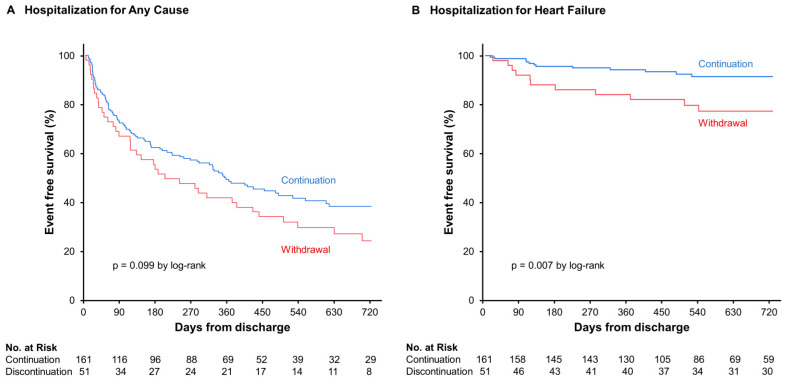
Hospitalizations for any cause (**A**) and recurrent heart failure (**B**).

Patients were followed for a median period of 695 (439–730) days. There were 93 readmissions (57.8%) in the continuation group and 38 readmissions (74.5%) in the withdrawal group. Patients in the withdrawal group tended to have a higher rate of hospitalization for any cause (*p* = 0.099; Figure 2A). The withdrawal of SGLT2i was not significantly associated with the primary outcome in the Cox analysis (*p* = 0.100). Instead, a history of atrial fibrillation (hazard ratio 1.44, 95% confidence interval 1.07–2.23), HFrEF (hazard ratio 0.66, 95% confidence interval 0.46–0.95), and eGFR (hazard ratio 0.99, 95% confidence interval 0.98–1.00) were independently associated with all-cause hospitalization (*p* < 0.050 for both; Table 2).

The number of hospitalization events for any cause was higher in the withdrawal group than in the continuation group (0.97 [0–1.50] versus 0.50 [0–1.03], *p* = 0.030). The multivariable analysis demonstrated that the withdrawal of SGLT2i (hazard ratio 1.41, 95% confidence interval 1.05–1.88) was independently associated with the number of readmission events for any cause, together with the higher prevalence of NYHA class III/IV symptoms (*p* < 0.050 for both; Table 3). The number of hospitalization events for non-cardiovascular disease was higher in the withdrawal group than in the continuation group (0 [0–0.69] versus 0 [0–0], *p* < 0.001). In contrast, the total number of cardiovascular events was comparable between both groups (0 [0–0.51] versus 0 [0–0.81], *p* = 0.941). Details of hospitalization events are listed in Supplementary Table S2.

### 3.3. Secondary Clinical Assessments

Patients in the withdrawal group had a higher rate of readmission for HF (21.6% versus 7.5%, *p* = 0.007; Figure 2B). The withdrawal of SGLT2i (hazard ratio 2.38, 95% confidence interval 1.04–5.56) and eGFR (hazard ratio 0.97, 95% confidence interval 0.94–0.99) were independently associated with readmission for HF (*p* < 0.050 for both; Table 4).

**Figure 3:**
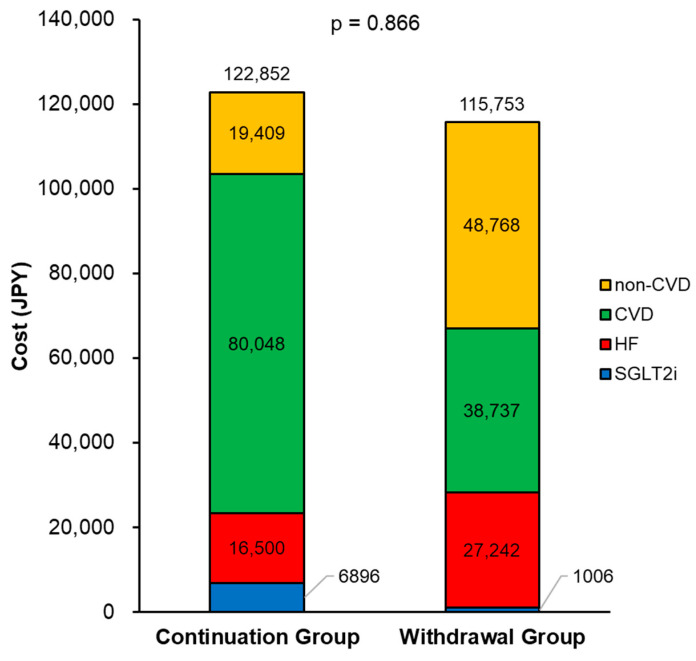
Breakdown of medical costs. JPY: Japanese yen; CVD: cardiovascular disease; HF: heart failure; SGLT2i: sodium–glucose cotransporter 2 inhibitors. The medical costs are expressed as the mean value.

In the withdrawal group, the median cost of SGLT2i was 153 (0–1747) JPY per month and the in-hospital medical cost was 62,906 (0–186,831) JPY per month. In the continuation group, the median cost of SGLT2i was 7920 (5640–7920) JPY and the in-hospital medical cost was 22,815 (0–172,385) JPY per month. There was no significant difference in total medical costs (62,906 [502–187,246] versus 29,236 [7920–180,305] JPY per month, *p* = 0.866; Figure 3) between the two groups.

## 4. Discussion

In this retrospective analysis, we investigated the impact of the withdrawal of SGLT2i after hospitalization for HF on clinical outcomes and cost-effectiveness. While the withdrawal of SGLT2i did not increase the risk of hospitalization for any cause, it did increase the total number of hospitalization events. Furthermore, the withdrawal of SGLT2i increased the risk of hospitalization for HF. There were no significant differences in medical costs between both groups (i.e., the withdrawal of SGLT2i did not have a significant benefit in reducing total medical costs).

### 4.1. Clinical Outcomes

In the additional trial after the end of the EMPEROR-Reduced and the EMPEROR-Preserved trials, patients withdrawn from treatment with empagliflozin showed an increased risk of HF events and worsening health status compared with patients withdrawn from the placebo [6]. Although the results of this trial indicate that the cessation of SGLT2i treatment may have deleterious consequences, some patients are unable to continue SGLT2i due to adverse events or chronic disease [16]. In this study, SGLT2i were terminated for reasons other than death in 51 patients (24.1%). Our withdrawal rate of 24.1% is higher than previously reported rates of 10.5–23.2% in RCTs [2,3,4,5]. Careful patient selection is mandatory in RCTs whereas we used real-world clinical data: our patients received SGLT2i soon after the episode of HF hospitalization, and their system conditions were relatively unstable.

In the present study, the withdrawal of SGLT2i increased the total number of all-cause hospitalization events, predominantly due to HF and non-cardiovascular events, whereas that of cardiovascular events was comparable. It is unclear as to why non-cardiovascular events increased in patients withdrawn from SGLT2i, but the underlying factors that led to the withdrawal of the drug may have also contributed to hospitalization events. Moreover, it is possible that its pleiotropic effects also suppressed not only HF events but also non-cardiovascular events.

We found that the withdrawal of SGLT2i increased the risk of hospitalization for HF. This is in line with the post-hoc analyses of the EMPEROR-Reduced and the EMPEROR-Preserved trials that the favorable effects of empagliflozin in patients with HF dissipated rapidly after withdrawal of the drug. On the other hand, participants in this study were observed for a longer period after the withdrawal of SGLT2i than in the previous trial, which was evaluated 30 days after withdrawal of the drug. Our finding suggests an increased risk of hospitalization events with long-term withdrawal of SGLT2i.

Unlike previous randomized trials, this observational study included patients withdrawn from SGLT2i for any reason. The reason for discontinuing SGLT2i might result in an increase in hospitalization events because there were different characteristics between patients who continued SGLT2i and those who discontinued them in this study. Nevertheless, multivariable analysis showed that the withdrawal of SGLT2i was the independent predictor of increasing the total number of hospitalization events. This finding demonstrates that the abrupt cessation of SGLT2i may have deleterious consequences for patients with HF, even if there is a plausible reason for the withdrawal of SGLT2i. However, the hospitalization events in this study included several elective cardiovascular or non-cardiovascular hospitalizations. Hence, it is somewhat challenging to assess the clinical significance of discontinuing SGLT2i with the findings of this study. Further research to facilitate the clinical importance of continuing SGLT2i is needed.

### 4.2. Medical Costs

Several cost-effectiveness analyses were performed using the Markov model which simulated the disease progression of HF patients with SGLT2i over their lifetime to capture all relevant costs and outcomes [17,18,19]. The model used estimates of treatment efficacy, event probabilities, and utilities from databases or published literature, whereas such analyses often do not model the adverse events caused by SGLT2i or their withdrawal. Minor adverse effects associated with SGLT2i, and their pleiotropic effects may make it difficult to identify their cost-effectiveness. Therefore, there has been no study evaluating the changes in actual medical costs associated with SGLT2i treatment. In further economic evaluations of SGLT2i for HF patients, epidemiological real-world data should be obtained. In our study, we investigated actual medical costs, including both in-hospital costs and SGLT2i costs, for the patients who received SGLT2i for the first time during their hospitalization for HF.

In this study, there was no significant difference in medical costs between patients with continuation of SGLT2i and those withdrawn from SGLT2i. Interestingly, in-hospital costs were similar despite differences in the number of hospitalization events between the two groups. The possible reasons for the uniformity of medical costs were mainly derived from the differences in causes of hospitalization. Patients withdrawn from SGLT2i had higher hospitalization costs for HF and those for non-cardiovascular disease than those with continuation of SGLT2i. In contrast, patients withdrawn from SGLT2 tended to have lower hospitalization costs for cardiovascular disease than those with continuation of SGLT2i. Consequently, medical costs for both groups were similar. This finding suggests that patients who continued SGLT2i may have undergone expensive treatments for cardiovascular disease (e.g., cardiac surgeries, cardiac devices, and transcatheter therapies). A possible explanation for the expensive treatments for patients with continuation of SGLT2i was that they were of a younger age, had higher body weight, or there was of a lower proportion of NYHA class III/IV at baseline. It was assumed to be the possibility that patients who were able to continue SGLT2i had a stable condition. Conversely, SGLT2i might stabilize patients with HF enough to undergo invasive treatments. Importantly, medical costs were consistent despite the differences in treatment details and clinical outcomes between the two groups.

### 4.3. Limitations

This study has the following limitations. First, this was an observational study conducted at a single center with a small sample size, not a randomized study. Given the low event number, the number of potential confounders included in the multivariable analyses was relatively restricted. Given the non-randomized selection, the lack of a control group prevents the true assessment of the adverse effects of discontinuing SGLT2i for HF patients. Also, it is not possible to establish a cause–effect relationship given the observational study design. A matched control assessment was abandoned due to the limited sample size. An inter-group comparison might have had selection bias with different background characteristics between the two groups. Specifically, patients withdrawn from SGLT2 were older and underweight. Nevertheless, the results for the primary clinical assessment remained significant in analysis with multiple imputations. Second, the timing of the withdrawal of SGLT2i varied depending on the participants in this study. Furthermore, it should be emphasized that other HF medications were also adjusted during the observational period. Thus, it is challenging to assess the clinical outcomes and the cost–benefits associated with a withdrawal of SGLT2i alone. Lastly, multiple types of SGLT2i including canagliflozin were used in the present study. No RCTs have evaluated the efficacy of canagliflozin in patients with HF. Nevertheless, a recent retrospective cohort study suggested that there was no significant difference in the risk of cardiovascular events including HF among patients taking dapagliflozin, canagliflozin, and empagliflozin [20]. On the other hand, a previous study demonstrated that empagliflozin was highly cost-effective compared with canagliflozin and dapagliflozin when using healthcare costs in the United States [21]. It remains unclear whether such cost-effectiveness is consistent across individual SGLT2i. Authors should discuss the results and how they can be interpreted from the perspective of previous studies and the working hypotheses. The findings and their implications should be discussed in the broadest context possible. Future research directions may also be highlighted.

## 5. Conclusions

In summary, the continuation of SGLT2i therapy for patients with HF reduced the total number of hospitalization events without increasing the medical cost burden.

## Figures and Tables

**Table 1:** Baseline characteristics at index discharge.

	Total (*n* = 212)	Continuation (*n* = 161)	Withdrawal (*n* = 51)	*p*-Value
Age, years	72 (66–81)	72 (63–79)	76 (71–84)	0.004 *
Male, *n* (%)	141 (67)	114 (71)	27 (53)	0.019 *
Body weight, kg	56.6 (49.9–67.5)	57.3 (50.9–69.3)	54.0 (44.1–64.1)	0.021 *
Body mass index, kg/m^2^	22.2 (19.8–24.8)	22.6 (20.2–25.3)	21.7 (18.8–23.6)	0.026 *
Systolic blood pressure, mmHg	107 (96–118)	107 (95–119)	106 (97–116)	0.990
Heart rate, beats per minute	71 (63–78)	70 (62–78)	71 (64–83)	0.772
Diabetes mellitus, *n* (%)	147 (69)	106 (66)	41 (80)	0.050 *
Ischemic etiology, *n* (%)	86 (41)	62 (39)	24 (47)	0.279
Atrial fibrillation, *n* (%)	61 (29)	43 (26)	18 (35)	0.238
Implantable cardioverter–defibrillator, *n* (%)	22 (10)	20 (12)	2 (4)	0.083
Cardio resynchronization therapy, *n* (%)	15 (7)	12 (8)	3 (6)	0.703
New York Heart Association class III-IV, *n* (%)	57 (27)	40 (25)	17 (33)	0.234
Left ventricular ejection fraction, %	43 (33–55)	44 (34–56)	42 (31–52)	0.294
Value of <40% (HFrEF), *n* (%)	84 (39)	59 (37)	25 (49)	0.115
HbA1c, %	6.6 (6.1–7.4)	6.6 (6.1–7.3)	6.7 (6.4–7.9)	0.115
Hemoglobin, g/dL	12.7 (11.2–14.2)	12.8 (11.5–14.3)	12.0 (10.8–13.8)	0.078
Serum albumin, g/dL	3.6 (3.3–3.9)	3.6 (3.4–3.9)	3.5 (3.1–3.7)	0.006 *
Serum sodium, mEq/L	138 (136–140)	138 (137–140)	137 (134–139)	<0.001 *
Serum potassium, mEq/L	4.4 (4.1–4.6)	4.4 (4.1–4.6)	4.4 (3.9–4.8)	0.612
eGFR, mL/minute/1.73 m^2^	51.1 (38.2–64.2)	52.8 (40.3–64.6)	49.2 (32.6–60.3)	0.109
Uric acid, mg/dL	5.7 (4.7–6.9)	5.5 (4.6–6.9)	5.9 (5.1–7.2)	0.060
Plasma BNP, pg/mL	142 (69–302)	132 (64–266)	182 (95–353)	0.022 *
Heart failure therapies				
Beta-blockers, *n* (%)	184 (87)	137 (85)	47 (92)	0.194
ACEI/ARB/ARNI, *n* (%)	200 (94)	153 (95)	47 (92)	0.439
Loop diuretics, n (%)	139 (66)	99 (62)	40 (78)	<0.001 *
Dose of loop diuretics, mg/day	10 (0–20)	10 (0–20)	20 (10–20)	0.223
MRA, *n* (%)	145 (68)	112 (70)	33 (65)	0.515
Sodium–glucose cotransporter 2 inhibitors				
Canagliflozin, *n* (%)	40 (19)	24 (15)	16 (31)	0.009 *
Dapagliflozin, *n* (%)	116 (55)	95 (59)	21 (41)	0.026 *
Empagliflozin, *n* (%)	56 (26)	42 (26)	14 (28)	0.847

eGFR, estimated glomerular filtration rate; HFrEF, heart failure with reduced ejection fraction (ejection fraction < 40%); HbA1c, glycated hemoglobin; BNP, b-type natriuretic peptide; ARB, angiotensin receptor blockers; ARNI, angiotensin receptor–neprilysin inhibitors; MRA, mineralocorticoid receptor antagonists. * *p* < 0.050.

**Table 2:** Variables associated with hospitalization for any cause.

	All Patients (*n* = 212)					
	Univariable Analysis			Multivariable Analysis		
Variables	Hazard Ratio	95% CI	*p*-Value	Hazard Ratio	95% CI	*p*-Value
Age, years	1.01	0.99, 1.02	0.394			
Male, yes	0.75	0.53, 1.07	0.112			
Body mass index, kg/m^2^	0.99	0.94, 1.03	0.510			
Systolic blood pressure, mmHg	1.00	0.99, 1.01	0.744			
Heart rate, bpm	1.01	0.99, 1.02	0.551			
Ischemic etiology, yes	0.99	0.70, 1.41	0.973			
Atrial fibrillation, yes	1.54	1.07, 2.21	0.022 *	1.44	1.07, 2.23	0.020 *
NYHA class III-IV, *n* (%)	1.29	0.88, 1.88	0.188			
HFrEF, yes	0.69	0.48, 0.99	0.041 *	0.66	0.46, 0.95	0.023 *
Diabetes mellitus, yes	1.36	0.92, 2.01	0.123			
Hemoglobin, g/dL	0.95	0.88, 1.03	0.239			
Serum albumin, g/dL	1.08	0.73, 1.58	0.713			
Serum sodium, mEq/L	1.00	0.95, 1.06	0.981			
Serum potassium, mEq/L	0.82	0.59, 1.15	0.238			
eGFR, mL/min/1.73 m^2^	0.99	0.98, 1.00	0.047 *	0.99	0.98, 1.00	0.044 *
Uric acid, mg/dL	0.98	0.88, 1.08	0.635			
ln BNP	1.08	0.91, 1.28	0.379			
Beta-blockers, yes	1.03	0.62, 1.72	0.905			
ACEI/ARB/ARNI, yes	0.70	0.37, 1.34	0.280			
Loop diuretics, yes	1.12	0.78, 1.62	0.531			
MRA, yes	0.81	0.56, 1.16	0.241			
Termination of SGLT2i, yes	1.37	0.94, 2.00	0.100			

NYHA, New York Heart Association; HFrEF, heart failure with reduced ejection fraction (ejection fraction < 40%); eGFR, estimated glomerular filtration rate; BNP, b-type natriuretic peptide; ACEI, angiotensin-converting enzyme inhibitors; ARB, angiotensin receptor blockers; ARNI, angiotensin receptor–neprilysin inhibitors; MRA, mineralocorticoid receptor antagonists. * *p* < 0.050.

**Table 3:** Variables associated with the total number of hospitalization events for any cause.

	All Patients (*n* = 212)					
	Univariable Analysis			Multivariable Analysis		
Variables	β	95% CI	*p*-Value	β	95% CI	*p*-Value
Age, years	−0.03	−0.06, 0.04	0.681			
Male, yes	−0.10	−1.07, 0.15	0.137			
Body mass index, kg/m^2^	−0.08	−0.23, 0.06	0.251			
Systolic blood pressure, mmHg	−0.08	−0.05, 0.02	0.280			
Heart rate, bpm	−0.05	−0.07, 0.03	0.530			
Ischemic etiology, yes	0.10	−0.14, 1.03	0.136			
Atrial fibrillation, yes	−0.03	0.79, 0.49	0.640			
NYHA class III-IV, n (%)	0.16	0.11, 1.40	0.022 *	0.15	0.06, 1.34	0.032 *
HFrEF, yes	0.06	−0.32, 0.86	0.369			
Diabetes mellitus, yes	−0.08	−0.99, 0.25	0.244			
Hemoglobin, g/dL	−0.04	−0.36, 0.20	0.568			
Serum albumin, g/dL	0.02	−1.08, 1.51	0.743			
Serum sodium, mEq/L	−0.04	−0.23, 0.12	0.559			
Serum potassium, mEq/L	0.08	−0.46, 1.89	0.230			
eGFR, mL/min/1.73 m^2^	−0.10	−0.05, 0.01	0.158			
Uric acid, mg/dL	0.01	−0.31, 0.34	0.923			
ln BNP	0.08	−0.22, 0.90	0.235			
Beta-blockers, yes	0.04	−0.63, 1.07	0.606			
ACEI/ARB/ARNI, yes	0.01	−1.18, 1.31	0.920			
Loop diuretics, yes	0.04	−0.41, 0.80	0.524			
MRA, yes	0.03	−0.51, 0.73	0.722			
Termination of SGLT2i, yes	0.15	1.40, 0.06	0.032 *	0.14	0.01, 1.34	0.048 *

NYHA, New York Heart Association; HFrEF, heart failure with reduced ejection fraction (ejection fraction < 40%); eGFR, estimated glomerular filtration rate; BNP, b-type natriuretic peptide; ACEI, angiotensin-converting enzyme inhibitors; ARB, angiotensin receptor blockers; ARNI, angiotensin receptor–neprilysin inhibitors; MRA, mineralocorticoid receptor antagonists. * *p* < 0.050.

**Table 4:** Variables associated with recurrent hospitalization for heart failure.

	All Patients (*n* = 212)					
	Univariable Analysis			Multivariable Analysis		
Variables	Hazard Ratio	95% CI	*p*-Value	Hazard Ratio	95% CI	*p*-Value
Age, years	1.01	0.97, 1.05	0.704			
Male, yes	0.93	0.40, 2.20	0.873			
Body mass index, kg/m^2^	0.98	0.87, 1.08	0.646			
Systolic blood pressure, mmHg	0.98	0.96, 1.01	0.230			
Heart rate, bpm	0.97	0.93, 1.01	0.115			
Ischemic etiology, yes	1.15	0.51, 2.63	0.733			
Atrial fibrillation, yes	2.34	1.03, 5.31	0.041 *	1.75	0.76, 4.03	0.193
NYHA class III-IV, n (%)	1.33	0.55, 3.25	0.526			
HFrEF, yes	1.11	0.49, 2.53	0.809			
Diabetes mellitus, yes	2.12	0.72, 6.24	0.171			
Hemoglobin, g/dL	0.88	0.71, 1.08	0.239			
Serum albumin, g/dL	0.87	0.35, 2.17	0.767			
Serum sodium, mEq/L	0.97	0.87, 1.10	0.641			
Serum potassium, mEq/L	0.75	0.36, 1.70	0.477			
eGFR, mL/min/1.73 m^2^	0.96	0.93, 0.99	0.003 *	0.97	0.94, 0.99	0.019 *
Uric acid, mg/dL	1.04	0.81, 1.30	0.751			
ln BNP	1.31	0.87, 2.08	0.222			
Beta-blockers, yes	1.02	0.30, 3.45	0.970			
ACEI/ARB/ARNI, yes	0.61	0.14, 2.60	0.502			
Loop diuretics, yes	3.61	1.07, 12.16	0.038 *	1.95	0.54, 7.04	0.308
MRA, yes	1.06	0.43, 2.56	0.907			
Termination of SGLT2i, yes	2.93	1.29, 6.65	0.010 *	2.38	1.04, 5.45	0.041 *

NYHA, New York Heart Association; HFrEF, heart failure with reduced ejection fraction (ejection fraction < 40%); eGFR, estimated glomerular filtration rate; BNP, b-type natriuretic peptide; ACEI, angiotensin-converting enzyme inhibitors; ARB, angiotensin receptor blockers; ARNI, angiotensin receptor–neprilysin inhibitors; MRA, mineralocorticoid receptor antagonists. * *p* < 0.050.

## Data Availability

The data presented in this study are available from the corresponding author upon request. The data are not publicly available due to privacy restrictions.

## Supplementary Materials

The following supporting information can be downloaded at: https://www.mdpi.com/article/10.3390/jcm13113196/s1. Table S1: Events leading to withdrawal of SGLT2 inhibitors; Table S2: Hospitalization events.
