# Supplementary material for: The Impact of the Withdrawal of SGLT2 Inhibitors on Clinical Outcomes in Patients with Heart Failure
Source: J Clin Med. 2024 May 29;13(11):3196. doi: 10.3390/jcm13113196 (PMC11172815; doi:10.3390/jcm13113196)
Supplement: Supplementary file 1 [file jcm-13-03196-s001.zip › jcm-2978983-supplementary.pdf]

Supplementary Materials

Table S1. Events leading to withdrawal of SGLT2 inhibitors.

	Number (%) of patients (n=51)
Urinary tract infection	8 (15.7)
Fasting	5 (9.8)
Hypotension	4 (7.8)
Regulation of blood glucose level	3 (5.9)
Self-discontinuation	3 (5.9)
Unknown	3 (5.9)
Weight loss	3 (5.9)
Bacterial infection	2 (3.9)
Dehydration	2 (3.9)
Diarrhea	2 (3.9)
Drug eruption	2 (3.9)
Liver dysfunction	2 (3.9)
Pneumonia	2 (3.9)
Pruritus	2 (3.9)
Swallowing dysfunction	2 (3.9)
Anorexia	1 (2.0)
Cardiac failure	1 (2.0)
Discretion of attending physician	1 (2.0)
Nausea	1 (2.0)
Poverty	1 (2.0)
Prescription failure	1 (2.0)

Table S2. Hospitalization events.

	Total number (%) of events (n=250)
Heart failure	38 (15.2)
Cardiac catheterization	28 (11.2)
Percutaneous coronary intervention	20 (8.0)
Cataract surgery	19 (7.6)
Catheter ablation for cardiac arrhythmia	19 (7.6)
Implantation of cardiac implantable electronic device	13 (5.2)
Transcatheter aortic valve implantation	10 (4.0)
Bacterial infection	8 (3.2)
Lung cancer	8 (3.2)
Gastrointestinal cancer	7 (2.8)
Valvular surgery	7 (2.8)
Abdominal surgery	6 (2.4)
Peripheral arterial disease	6 (2.4)
Cholecystitis	5 (2.0)
Orthopedic disease	5 (2.0)
Pneumonia	5 (2.0)
Coronary artery bypass graft surgery	4 (1.6)
Gastrointestinal bleeding	3 (1.2)
Cerebral infarction	3 (1.2)
Diabetes	3 (1.2)
Aortic disease	2 (0.8)
Arrythmia	2 (0.8)
Cholesterol crystal embolization	2 (0.8)

Hematoma	2 (0.8)
multiple myeloma	2 (0.8)
Prostate disease	2 (0.8)
Transcatheter mitral valve repair	2 (0.8)
Acute kidney injury	1 (0.4)
Balloon pulmonary angioplasty	1 (0.4)
COVID-19 infection	1 (0.4)
Deafness	1 (0.4)
Dehydration	1 (0.4)
Diabetic ketoacidosis	1 (0.4)
Endoscopic polypectomy	1 (0.4)
Frailty	1 (0.4)
Gastrointestinal dysfunction	1 (0.4)
Herpes zoster	1 (0.4)
Implantation of interatrial shunt device	1 (0.4)
Lymphoma	1 (0.4)
Percutaneous left atrial appendage closure	1 (0.4)
Pulmonary embolism	1 (0.4)
Sarcoidosis	1 (0.4)
Skin cancer	1 (0.4)
Skin graft surgery	1 (0.4)
Suffocation	1 (0.4)
Urinary tract infection	1 (0.4)
